# Notes and News

**Published:** 1897-01-16

**Authors:** 


### Notes and News.

(Collected and Communicated.)

Professor Von Pettenhofer, Emeritus Professor of Hygiene at Munich, has received the Harben Medal for 1897 from the Council of the British Institute of Public Health.

H.R.H. the Duke of Connaught will preside at the Biennial Festival of the National Hospital for the Paralysed and Epileptic (Albany Memorial), Queen Square, Bloomsbury, on March 24th next.

The French President continues to take great interest in charitable institutions. He recently visited the Ivry Hospital, which shelters upwards of 800 old and incurable women. Some of the inmates have attained a great age, one woman of 99 years being a great-great-great-grandmother.

Among the troubles brought upon Cuba by the rebellion is a very large increase in the prevalence of yellow fever, due, no doubt, to the introduction of numbers of soldiers from Europe who were unacclimatised. Like other infectious diseases, yellow fever increases much in virulence in the presence of large numbers of susceptible people.

It is to be hoped that the occupation of the new temporary buildings, one block of which is now completed, will cause some abatement of the epidemic of Beri-Beri, which is still prevalent at the Richmond Asylum, Dublin. There is undoubted overcrowding, which has probably retarded the extermination of the disease.

It has unfortunately been found necessary to establish a national fund for the aid of the sufferers from the Indian famine. The appeal emanates from the Mansion House, and the name of the Queen Empress appropriately heads the donation list with a subscription of £500. The close connection between the British and the Indian Empire is more apparent every year; and on the present occasion evidence is shown that not only the Queen as Empress of India is ready to hasten to the aid of the sufferers in India, but that the British workman is responding with sympathy and generosity to the cry for aid from their fellow-subjects.

Mr. Bancroft will give one of his popular readings of Dickens, on behalf of the Bristol Royal Infirmary, on January 28th.

Some of the large firms have been very liberal in responding to the appeal for the new infirmary at Newcastle. The latest donation from this source is a gift of £1,000 from the Newcastle Breweries Company.

Professor Koch paid a visit to Robben Island to inspect the Leper Colony, before proceeding to Kimberley. He expressed admiration for the conduct of the place, and congratulated the Government and the officials.

We are glad to learn that the Alexandra Hospital for Children with Hip Disease has received donations recently amounting to £400. The small hospital is carried on in a very unostentatious manner and in the face of great difficulties, owing to its crowded site. The hospital is really a "home," as well as a hospital, for the necessary residence of the little patients is seldom a short one. It is, therefore, pleasing to witness the content which prevails, and how much is done to brighten tedious hours, and cause the children to forget the limited movement which it is necessary in most cases to enforce.

Our Indian Empire is passing through a tide of misfortune at the present time. With all the horrors of famine to face there is yet another check to the industries of the country through the plague at Bombay, which unfortunately shows no abatement. There is great panic amongst native hands, and in the cotton and other mill industries the falling off in production is reported to equal 75 per cent. News that the plague has spread in some degree amongst Europeans in Bombay has aroused a considerable feeling of alarm, and the loss of Dr. Manser, the first English victim, has been a great shock. Dr. Manser was, perhaps, the most popular doctor in Bombay, and he will be much missed. The precautionary sanitary measures are not understood by the natives, and no confidence is felt in them, This is, of course, one of the greatest difficulties the authorities have to face.

The report of the arrival of the cholera-stricken ship "Nubia" at Plymouth has created a great sensation, especially locally. The "Nubia" is a troopship from India, and the cholera first showed itself at Malta. So far four of the troops have succumbed to the disease. Immediately on her arrival at Plymouth, the "Nubia" was boarded by the medical officers of health. Fortunately the medical arrangements at Plymouth are good. There are two hospital ships, and to one of these the mild cases have been removed. Not a little alarm was felt in some quarters when it was remembered that the quarantine regulations have been repealed; but this alarm should be unnecessary, seeing that the medical officer at every port has full authority to take any steps for the safety of the public that he may deem needful. The position of the medical officer is, therefore, a strong one. Promptness action is facilitated, and the grave responsibility which is personally vested in him tends to make the officer more and not less vigilant to carry out protective measures.

